# How to Achieve High-Quality Oocytes? The Key Role of Myo-Inositol and Melatonin

**DOI:** 10.1155/2016/4987436

**Published:** 2016-08-29

**Authors:** Salvatore Giovanni Vitale, Paola Rossetti, Francesco Corrado, Agnese Maria Chiara Rapisarda, Sandro La Vignera, Rosita Angela Condorelli, Gaetano Valenti, Fabrizio Sapia, Antonio Simone Laganà, Massimo Buscema

**Affiliations:** ^1^Unit of Gynecology and Obstetrics, Department of Human Pathology in Adulthood and Childhood “G. Barresi”, University of Messina, Messina, Italy; ^2^Unit of Diabetology and Endocrino-Metabolic Diseases, Hospital for Emergency Cannizzaro, Catania, Italy; ^3^Department of General Surgery and Medical Surgical Specialties, University of Catania, Catania, Italy; ^4^Department of Clinical and Experimental Medicine, Research Centre of Motor Activity and Metabolic Rehabilitation in Diabetes (CRAMD), University of Catania, Catania, Italy

## Abstract

Assisted reproductive technologies (ART) have experienced growing interest from infertile patients seeking to become pregnant. The quality of oocytes plays a pivotal role in determining ART outcomes. Although many authors have studied how supplementation therapy may affect this important parameter for both in vivo and in vitro models, data are not yet robust enough to support firm conclusions. Regarding this last point, in this review our objective has been to evaluate the state of the art regarding supplementation with melatonin and myo-inositol in order to improve oocyte quality during ART. On the one hand, the antioxidant effect of melatonin is well known as being useful during ovulation and oocyte incubation, two occasions with a high level of oxidative stress. On the other hand, myo-inositol is important in cellular structure and in cellular signaling pathways. Our analysis suggests that the use of these two molecules may significantly improve the quality of oocytes and the quality of embryos: melatonin seems to raise the fertilization rate, and myo-inositol improves the pregnancy rate, although all published studies do not fully agree with these conclusions. However, previous studies have demonstrated that cotreatment improves these results compared with melatonin alone or myo-inositol alone. We recommend that further studies be performed in order to confirm these positive outcomes in routine ART treatment.

## 1. Introduction

Female infertility, defined as a failure to achieve a successful pregnancy after 12 months or more of appropriate, timed unprotected intercourse or therapeutic donor insemination [1], has various causes and elements that play a key role including, aging, acute and chronic diseases, pharmacological therapies, behavioral factors, and exposure to environmental, occupational, and infectious agents. Data from 2006 to 2010 from the Center for Disease Control (CDC) National Survey of Family Growth show that, among all married US women aged 15–44 years, 6% (1.5 million women) were infertile and 12% (3.1 million women) had impaired fertility, defined as a condition in which there is physical difficulty in getting pregnant or carrying pregnancy to term [2]. These figures would be even more alarming if the data included statistics regarding couples who tried for one year or more to become pregnant and failed [3].

In recent decades, maternal age has progressively increased: in 1975 only 5% of women who became pregnant were over 30 years old, whereas in 2010 this percentage was 26% [4, 5]. Aging is one of the principal factors related to female fertility, with a direct correlation between increased maternal age and infertility/subfertility rates, probably due to diminished egg quality, ovulatory dysfunction, and reproductive disorders such as tubal diseases, leiomyoma, and endometriosis [6]. According to recent data, the fertility decline begins at the middle of third decade [7]. Reproductive outcomes depend directly on the quality of oocytes, which develop proper competence according to their genetic and epigenetic programming. The integrity of cytoskeleton, mitochondria function, and distribution play a pivotal role during the spindle formation and consequently modulate chromosomal segregation and maintain genome stability after division [8, 9]. Spindle is composed of bundles of microtubules, which are polar polymers of *α*- and *β*-tubulin heterodimers. The most common defect in aging is shortening and disorganization of spindle, and this alteration may predispose the oocyte to aneuploidy or maturation arrest. In addition, mitochondria and endoplasmic reticulum control calcium-mediated intracellular signaling. This is of paramount importance for oocyte activation and modulates calcium/calmodulin-dependent protein kinase II, which is implicated in the transition from metaphase I to anaphase I [8].

Currently many infertile women undergo assisted reproductive technologies (ART) [10], dating from the first baby born after in vitro fertilization (IVF) and embryo transfer (ET) [11]. Thereafter, women affected by primary ovarian failure became pregnant with egg donation [12], a common procedure today [13]. Other techniques have been developed, such as gamete intrafallopian transfer (GIFT) and zygote intrafallopian transfer (ZIFT), although these are not universally applicable, for example, for women with tubal occlusion or in the case of male infertility [10]. ART has continued to grow with improvements in ultrasonography, including ultrasound-guided transvaginal follicular aspiration [14] with a consequent reduction in women's health risks and expenses. Intracytoplasmic sperm injection (ICSI) [15] was a step forward which improved outcome in the case of male infertility. Today IVF represents 99.5% of ART [13]. With growing interest in IVF as an answer to infertility, the interest of the scientific community is focused on supplementation, which can improve the outcome of these techniques.

## 2. Rationale for the Use of Melatonin

In recent years, attention has been focused on the negative impact of oxidative stress due to an excess of free radicals on oocyte quality during the IVF cycle. Free radicals are represented by reactive oxygen species (ROS), the most important class generated in living systems [16] and reactive nitrogen species (RNS). Free radicals play a dual role with both deleterious and beneficial effects. Physiologically free radicals maintain a balance in cells, called “redox balance” according to both its production and antioxidant cellular capacity. These are represented by both enzymatic antioxidant defenses, such as superoxide dismutase (SOD), glutathione peroxidase (GPx), catalase (CAT), and nonenzymatic antioxidant defenses, like ascorbic acid, *α*-tocopherol, glutathione (GSH), carotenoids, flavonoids [17], and other molecules such as melatonin.

The overproduction of ROS results in oxidative stress, which can be an important mediator of damage to cell structures as lipids, membranes, proteins, and DNA [18]. On the other hand, free radicals are involved in cellular signaling pathways, in the induction of mitosis, and in defense against* noxa* as infectious agents. One physiological function in which ROS plays an fundamental role is reproductive physiology, including follicular development, oocyte maturation, ovulation, corpus luteum function, and follicular atresia [19]. Several authors compared ovulation to an inflammatory reaction with the production of cytokines and free radicals [19, 20]. The follicular fluid (FF) obtained from antrum of Graafian follicles has a higher melatonin concentration than a plasma sample collected simultaneously [21]. This is probably related to the necessity to protect the oocyte from free radicals formed during oocyte maturation and to preserve oocyte quality [22]. As support, one study indicated that reduced antioxidant enzyme level was reported in the FF of women with unexplained infertility [23].

In 1993 it was discovered that melatonin is a potent endogenous scavenger of free radicals [24]. Before then melatonin was known as a hormone, produced by the pineal gland, with the ability to modulate circadian and reproductive physiology in photoperiod-dependent, seasonally breeding mammals [25]. Today it is known that melatonin links its receptors in the suprachiasmatic nuclei of the brain and in pars tuberalis in the pituitary gland to modulate the reproductive function. The presence of melatonin receptors is found in many other tissues of the reproductive system (epididymis, vas deferens, prostate, ovary, and mammary gland) [26] and other tissues including the skin, gastrointestinal tract, liver, kidney, spleen, and immune system cells. Enzymes involved in melatonin synthesis have been found in most of these tissues. These findings may relate to a hypothesis of multiple functions performed by melatonin (antioxidant, anti-inflammatory properties, genomic stabilizing effects, and modulator of mitochondrial homeostasis) [27].

Melatonin acts directly as a scavenging free radical [28] or in binding with its receptors [29] to prevent damage in cells and tissues. The antioxidant melatonin mechanism is different from other antioxidants because, after reacting with free radicals in a “scavenging cascade reaction,” it generates several stable antioxidant end-products. It does not promote oxidation under any circumstances as this would terminate its properties as an antioxidant. In addition, melatonin synergizes with other nonenzymatic antioxidants [30] and also stimulates antioxidant enzyme synthesis by binding its membrane receptors to cytosolic or nuclear-binding sites [31].

Tamura et al. [32] measured melatonin concentration in FF in humans during oocyte retrieval for IVF-ET and correlated it with levels of 8-hydroxy-2-deoxyguanosine (8-OHdG), which is a product of free radical damage. They found an inverse correlation between two molecules, suggesting that 8-OHdG is an index of degeneration of oocytes. Furthermore, the inverse relation between melatonin and 8-OHdG may confirm the protective role of melatonin against oxidative stress [32]. In line with this last finding, a recent study [33] showed that mature follicles have a higher level of melatonin compared to immature follicles and that melatonin is secreted from luteinizing granulosa cells (GC). The study measured mRNA of enzymes involved in melatonin synthesis and found increased levels of mRNA in GC, which were able to be synthesized by this hormone. Based on these data, it is possible to speculate that melatonin may be released from luteinizing GC during late folliculogenesis and is involved in oocyte maturation.

During IVF, oocyte and embryos may be exposed to high levels of superoxide free radicals started during the stimulation protocol, in which oocyte manipulation alters the level of endogenous oxygen scavengers [34]. FF rich in antioxidants is absent in oocyte medium cultures including melatonin, which protects oocyte from oxidative stress. Furthermore the ROS level is higher during IVF due to a high oxygen concentration level in incubators and handing throughout the IVF process [35]. With the increase in “oxidative stress” during IVF, melatonin, with its scavenger properties, finds its proper use.

## 3. Use of Melatonin in ART

It was observed that adding melatonin in mouse oocyte cultures resulted in 54% of fertilized oocytes that reached the blastocyst stage compared to 29% without melatonin [36]. Other authors studied porcine oocytes cultures and found that adding melatonin to oocyte culture media produced a significantly lower level of ROS and a greater proportion of mature oocytes (MII) [37]. Recent evidence suggests that a concentration of melatonin in oocyte culture should be in a narrow range between 10^−6^ and 10^−9^ nM to achieve the best results.

Some authors found that in humans supplementation with a low dose of melatonin improved oocyte nuclear maturation, and conversely decreased nuclear maturation was observed with a high dose [38]. A range from 10^−5^ to 10^−9^ nM improved oocyte maturation when added at in vitro oocyte maturation (IVM), and it augmented the implantation rate during IVF-ET, increasing the pregnancy rate in women with PCOS, although in some cases the increase was not significant [33].

Tamura et al. [32], in addition, showed that oral administration of melatonin raised the concentration of melatonin in FF, and this higher concentration was inversely related to cellular oxidative stress measured by the 8-OHdG level. In particular, they studied patients who had undergone unsuccessful IVF, dividing patients into two groups: the first treated with orally administered melatonin (3 mg/day) and the second without therapy. Considering their data analysis, they found that the first group had a higher fertilization rate than second group.

Other studies show that melatonin significantly improved morphologically MII during IVF compared to patients not treated. In contrast to Tamura et al.'s data [32], these studies did not find an increased fertilization rate, although the number of class 1 embryos was higher in the treated group [39, 40], and pregnancy rates were higher in the treated group although the difference did not reach statistical significance [39].

Other authors showed that the fertilization rate improved with melatonin supplementation both in those with a lower fertilization rate during a previous IVF cycle and in those who underwent an ICSI procedure [41]. For a complete summary of the studies reported in this chapter, refer to Table 1.

## 4. Rationale for the Use of Myo-Inositol

Inositols are multifaceted molecules that play a fundamental role in many cellular functions. Myo-inositol (MI) and D-chiro-inositol (DCI) are two stereoisomers of a six-carbon cyclitol ring, and MI is a precursor of DCI and is most widely distributed in nature. Inositol is assumed to be an essential B complex vitamin with its main source being a normal diet, although it can be synthetized in the human. Inositols may be present in cells in different ways: incorporated in membrane phospholipids as phosphoinositides [42] and in the form of inositol phosphoglycans (IPG) located in the cytoplasmic membrane, which are involved in insulin transduction signaling as secondary messengers [43]. The role of phosphoinositides has long been known. They are fundamental elements of the cellular membrane, where inositol combines with cytidine diphosphate diacylglycerol to form phosphatidylinositol (PtdIns), a fundamental constituent of cell membranes. Inositol is involved in the phosphoinositides signaling pathway and plays a major role in numerous cellular functions [42]: phosphatidylinositol 4P (PtlIns-4P) and phosphatidylinositol 4,5 bisphosphate (PtdIns 4,5P) are the most studied. Two important second intracellular messengers, diacylglycerol (DAG) and inositol-triphosphate (Ins 1,4,5-P), are produced from PtdIns 4,5P. Ins 1,4,5-P binds Ca2+ channels in the rough endoplasmic reticulum stimulating intracellular Ca2+ release and, subsequently, numerous related intracellular effects. The increase in intracellular Ca2+ levels plays an important role in oocyte maturation, fertilization, and embryo development [44–46]. Intracellular calcium-release mechanisms are modulated during the oocyte maturation process, particularly during the final stages of oocyte maturation. A major sensitivity to calcium release was observed in the final stage [47].

For all these reason, MI is involved in phosphoinositides pathway signaling and plays a key role in oocyte fertilization: many studies describe the relationship of its concentration level in FF, oocyte quality, and estrogen level in FF both in* in vitro* mouse models and in humans during IVF procedures [48, 49].

PtdIns 4,5P is involved in the development of cytoskeleton (CSK) and is involved in the binding between actin and the cellular membrane [42], suggesting a protective function for nuclear chromatin [50]. Another member of PtdIns molecules, PtdIns 3,4,5P, is involved in nuclear modulation of transcriptosome [51]. Some authors speculate that the depletion of inositol may desensitize the PtdIns pathway by slowing down resynthesis of a precursor [52].

Another important aspect of inositols is their role as insulin-sensitizers. In the form of IPG, they are able to activate insulin signaling pathways. IPG binds insulin with G-protein to create a coupled receptor to act as a secondary messenger, that is, DCI-phosphoglycan, which allows the activation of glycogen synthase both directly and indirectly via phosphatidylinositol 3-kinase (PIK3). It works to activate pyruvate dehydrogenase inside the mitochondria controlling oxidative glucose metabolism [53]. Conversely MI-phosphoglycan, based on IPG, allows the inhibition of cyclic adenosine monophosphate (cAMP) kinase and adenylate cyclase that are a regulatory enzyme in free fatty acid (FFA) metabolism [54].

Previous data analysis [55] showed that the DCI level was 50% lower in muscle and urine samples derived from type II diabetic patients compared with samples from control subjects. Conversely MI did not differ between two groups studied. The imbalance between MI and DCI levels was called “inositol imbalance.” The decline in DCI levels and inositol imbalance can be linked to insulin resistance (IR); and DCI levels worsened with the increase of insulin-resistance level [56, 57].

Many studies have shown that in patients with insulin resistance, such as in PCOS patients, using DCI, MI, and MI plus DCI may improve insulin resistance, metabolic parameters, regularity of cycle, and spontaneous ovulation [58–63] in keeping with insulin-sensitizer effects of DCI and the role of MI in oocytes maturation.

However a recent study confirmed that high DCI dosage (higher than 2.4 g/daily) had a negative effect on IVF outcome [64]. These data may explain the DCI ovary paradox theory in which insulin sensitivity remained in IR ovarian cells with a hyperstimulated epimerase function and with an imbalance between lower MI and higher DCI changing the physiologic MI/DCI ratio [65].

## 5. Use of Myo-Inositol in ART

It has been suggested that MI added to in vitro culture media may increase bovine blastocyst development [66] just as MI improved in vitro maturation in mouse oocytes [67].

Chiu et al. [48] divided into two groups, according to oocytes quality, the oocytes retrieved from women treated with IVF: in group A they included good quality oocytes, whereas in group B they included bad quality oocytes. They showed that group A had significantly higher FF volume and higher MI level in FF without any difference in MI serum level between the two groups; MI was found to be higher in FF with oocytes that developed embryos with good morphology, with a positive correlation between MI level and fertilization rate. Also the estradiol level was higher in FF than in serum and was positively correlated with MI level in FF. Based on this evidence, a higher concentration of MI in FF could be due to the active transport of MI into FF as previously shown in an animal model [67]. These data suggest that MI is required to enhance the development of the maturing oocyte [48]. Other authors have shown that women under 40 years old with at least one of following features: a previous failed attempt with ICSI with low quality oocytes, or PCOS, or a diagnosis of “poor responders,” treated with MI plus folic acid had higher number of good quality oocytes versus patients treated with only folic acid. The number of grade A embryos significantly increased the clinical pregnancy rate without reaching a significant difference if clinical and biochemical pregnancy rates are considered [68]. Another study confirmed the positive role of MI plus folic acid supplementation versus only folic acid in patients with PCOS, increasing the number of oocytes recovered and increasing the numbers of ET and good quality embryos while at the same time a reduction of immature oocytes was observed [69, 70]. MI used in women with PCOS caused a reduction in the total number of units and number of days of FSH stimulation, reducing estradiol (E2) levels measured the day before human chorionic gonadotropin (hCG) administration, allowing a decreased risk of hyperstimulation syndrome [70]. Other authors compared MI with DCI in patients, finding that MI had the capacity to ameliorate the quality of oocytes, to decrease the number of immature oocytes, and to improve the number of good quality embryos and the total number of pregnancies compared to DCI treatment during an ovarian stimulation protocol [71]. Other authors [72] found that MI plus DCI improved the quality of oocytes and the quality of embryos, reducing immature oocytes compared to only DCI treatment both in women under and over 35 years old. In patients under 35 years old, MI plus DCI also improved the fertilization rate. These authors speculated that MI plus DCI together can more quickly improve “inositol imbalance” and that DCI alone should be avoided in the IVF technique. For a complete summary of the studies reported in this chapter, refer to Table 2.

## 6. Myo-Inositol and Melatonin in ART

Recently many authors have investigated whether supplementation with MI and melatonin (plus folic acid), administered together, improved the PCOS outcome in IVF. These data suggested a synergistic effect with both MI and melatonin, which were able to improve the number of MII and the number of grade I embryos (good quality) compared to MI alone [73, 74]. Furthermore, MI and melatonin reduced significantly the units of gonadotropin used for stimulation as well as the E2 level reached before hCG injection. There was no significant increase in pregnancy rates in the study group with respect to controls [73], although others [74] found that both the fertilization rate and the pregnancy rate tended to be higher in the group cotreated with melatonin. Based on these data, it is possible to speculate that the positive effect of melatonin as ROS scavengers and its effect on the increase in LH receptors and progesterone synthesis bound its receptor in granulosa-luteal cells resulting in luteinization and ovulation. MI also plays a crucial role in oocyte maturation and as an insulin-sensitizer molecule. These features of MI and melatonin lead to a positive effect on oocyte and embryo quality in IVF [73]. In addition, therapy with MI plus M in patients who failed to conceive in previous IVF cycles due to poor oocyte quality has been shown to improve oocytes and embryo quality during second IVF cycle, with a tendency toward augmented pregnancy rates, although it did not reach statistical significance in a large-cohort analysis [75]. For a complete summary of the studies reported in this chapter, refer to Table 3.

## 7. Conclusion

Melatonin is an oxidative scavenger and has an important role in the reduction of oxidative stress which physiologically increases during ovulation. This effect becomes more important during IVF. The manipulation of oocytes, incubated with high oxygen levels, increases the oxidative stress. Studies in vitro and in vivo have shown that melatonin improved oocyte quality and embryo quality. Some studies have shown an increased fertilization rate, and in one study it increased the implantation rate. The pregnancy rate tended to increase, although it was not statistically significant. As expected, melatonin treatment increased the melatonin concentration in FF and reduced the oxidative stress in FF measured by the 8-OHdG level.

MI plays an important role in cytoskeleton and in chromatin stabilization, all necessary features for correct oocyte maturation. It plays an important role as a PtdIns precursor involved in the intracellular Ca2+ signaling pathway that has been shown to be fundamental to proper oocytes maturation. Furthermore inositols have an insulin-sensitizer effect. According to our overview, MI improves oocyte and embryo quality in patients undergoing IVF and is able to reduce units and days of FSH stimulation and E2 levels before hCG administration, thus reducing the risk of hyperstimulation syndrome especially in patients with PCOS and thus increasing pregnancy rates.

Combined therapy with MI and melatonin increased the positive effects described previously on outcome of ART. Cotreated patients showed an improved number of good quality oocytes and embryos, with reduced FSH units and days of treatment during cycles IVF.

Although these results are positive, more studies in large populations are necessary to confirm the data and to support the use of this supplementation therapy routinely in ART.

## Figures and Tables

**Table 1:** Effects of melatonin supplementation therapy (adding in oocytes culture or orally administered to patients) during ART.

Authors	Species	Melatonin in vitro/in vivo	Patients number (tot)	Intervention/control number	Technique	Significant outcomes (*p* < 0.05)
Lord et al. 2013 [36]	Mouse	Adding M in oocyte culture medium				(i) Increased number of oocytes that reached blastocyst stage

Kang et al. 2009 [37]	Porcine	Adding M in oocyte culture medium				(i) Increased mature oocyte

Tamura et al. 2008 [32]	Human	Orally administered M (3 mg/day)	115	56 versus 56 nontreated 1° cycle versus treated 2° cycle	IVF-ET	(i) Improved fertilization rate
		M (3 mg/day) versus Vit. E (600 mg/day) versus M + Vit. E (3 mg + 600 mg/day)	18	59 versus 59 nontreated 1° cycle versus nontreated 2° cycle		(i) Reduced 8-OHdG in FF (ii) Increased in FF in treated patient.

Kim et al. 2013 [33]	Human	Adding M in oocyte culture medium	111	62/49	IVM IVF-ET	(i) Increased mature oocyte (ii) Increased implantation

Batioglu et al. 2012 [39]	Human	Orally administered M (3 mg/day)	85	40/45	IVF-ET	(i) Increased mature oocyte (ii) Increased good quality embryos

Eryilmaz et al. 2011 [40]	Human	Orally administered M (3 mg/day)	60	30/30	IVF-ET	(i) Increased mature oocyte (ii) Increased good quality embryos

Nishihara et al. 2014 [41]	Human	Orally administered M (3 mg/day)	97	97/97 (control of itself)	IVF ICSI	(i) Improved fertilization with ICSI (ii) Improved fertilization in poor responder (iii) Improved good quality embryos

M: melatonin; Vit. E: vitamin E; IVF: in vitro fertilization; ET: embryo transfer; IVM: in vitro maturation; ICSI: intracytoplasmic sperm injection; 8-OHdG: 8-hydroxy-2-deoxyguanosine.

**Table 2:** Effects of myo-inositol supplementation therapy (adding in oocytes culture or orally administered to patients) during ART.

Authors	Species	Myo-inositol in vitro/in vivo	Patients number (tot)	Intervention/control number	Technique	Significant outcomes (*p* < 0.05)
Pesty et al. 1994 [67]	Mouse	Adding MI in oocyte culture medium				(i) Improved oocyte maturation

Chiu et al. 2002 [48]	Human	Adding MI in oocyte culture medium	53	32/21	IVF	(i) Improved good quality oocyte (ii) Increased FF volume

Brusco and Mariani 2013 [68]	Human	Orally administered MI + FA (2 g + 400 mg/day) versus FA (400 mg/day)	149	58/91	IVF-ET ICSI	(i) Improved number of good quality oocytes (ii) Improved number embryos of grade A (iii) Increased clinical pregnancy rate

Ciotta et al. 2011 [69]	Human	Orally administered MI + FA (2 g + 200 mg/twice daily) versus FA (400 mg/day)	34	17/17	IVF-ET ICSI	(i) Increased number oocytes retrieved (ii) Increased numbers embryos transfer (iii) Increased grade S1 embryos (iv) Reduced number of immature oocytes

Unfer et al. 2011 [71]	Human	Orally administered MI (2 g/twice daily) versus DCI (0.6 g/twice daily)	84	43/41	IVF-ET ICSI	(i) Increased numbers mature oocyte (ii) Reduced number of immature oocytes (iii) Increased embryos grade 1

Colazingari et al. 2013 [72]	Human	Orally administered MI + DCI (1.1 g + 27.6 mg/day) versus DCI (0.5 g/day)	100	46/53	IVF-ET	(i) Improved oocyte quality (ii) Improved embryos quality (iii) Improved pregnancies rate

Papaleo et al. 2009 [70]	Human	Orally administered MI + FA (2 g + 200 mg/twice daily) versus FA (400 mg/day)	60	30/30	IVF-ET ICSI	(i) Reduced number of immature oocytes (ii) Decreased FSH unit and day of stimulation (iii) Decreased E2 level before hCG stimulation

MI: myo-inositol; FA: folic acid; E2: estradiol; DCI: D-chiro-inositol; FF: follicular fluid; IVF: in vitro fertilization; ET: embryo transfer; ICSI: intracytoplasmic sperm injection; FSH: follicle stimulating hormone; hCG: human chorionic gonadotropin.

**Table 3:** Effects of myo-inositol + melatonin supplementation during ART.

Authors	Species	Myo-inositol + melatonin in vitro/in vivo	Patients number (tot)	Intervention/control number	Technique	Significant outcomes (*p* < 0.05)
Pacchiarotti et al. 2016 [73]	Human	Orally administered MI (4 g/day) + FA (400 mcg/day) + M (3 g/day) versus MI (4 g/day) + FA (400 mcg/day) versus FA (400 mcg/day)	526	165/166/195	ICSI IVF-ET	(i) Increased mature oocytes (ii) Increased embryos grade 1 (iii) Reduced days of FSH stimulation (iv) Reduced E2 level before hCG

Unfer et al. 2011 [75]	Human	Orally administered MI (4 g/day) + FA (400 mcg/day) + M (3 g/day) versus previous failed IVF (themselves)	46	46/46	IVF-ET	(i) Increased mature oocytes (MII) (ii) Increased embryos grade 1 (iii) Reduced days and UI of FSH stimulation

Rizzo et al. 2010 [74]	Human	Orally administered MI (4 g/day) + FA (400 mcg/day) + M (3 g/day) versus MI (4 g/day) + FA (400 mcg/day)	65	32/33		(i) Increased mature oocytes (MII) (ii) Increased embryos grade 1-2

MI: myo-inositol; FA: folic acid; M: melatonin; IVF: in vitro fertilization; ET: embryo transfer; ICSI: intracytoplasmic sperm injection; FSH: follicle stimulating hormone; hCG: human chorionic gonadotropin; UI: uterine insemination.
